# Gut microbiota derived metabolites contribute to intestinal barrier maturation at the suckling-to-weaning transition

**DOI:** 10.1080/19490976.2020.1747335

**Published:** 2020-04-30

**Authors:** Martin Beaumont, Charlotte Paës, Eloïse Mussard, Christelle Knudsen, Laurent Cauquil, Patrick Aymard, Céline Barilly, Béatrice Gabinaud, Olivier Zemb, Sandra Fourre, Roselyne Gautier, Corinne Lencina, Hélène Eutamène, Vassilia Theodorou, Cécile Canlet, Sylvie Combes

**Affiliations:** a GenPhySE, Université De Toulouse, INRAE, ENVT, Toulouse, France; b GEC Consortium CCPA, Evialis, Inzo, MixScience, Techna, Toulouse, France; c GeT-PlaGe, Genotoul, INRAE, Toulouse, France; d Toxalim (Research Centre in Food Toxicology), Université De Toulouse, INRAE, ENVT, INP-Purpan, UPS, Toulouse, France

**Keywords:** Early life, intestinal barrier development, organoids, epithelium, maternal milk, solid food, metabolome

## Abstract

In suckling mammals, the onset of solid food ingestion is coincident with the maturation of the gut barrier. This ontogenic process is driven by the colonization of the intestine by the microbiota. However, the mechanisms underlying the microbial regulation of the intestinal development in early life are not fully understood. Here, we studied the co-maturation of the microbiota (composition and metabolic activity) and of the gut barrier at the suckling-to-weaning transition by using a combination of experiments *in vivo* (suckling rabbit model), *ex vivo* (Ussing chambers) and *in vitro* (epithelial cell lines and organoids). The microbiota composition, its metabolic activity, para-cellular epithelial permeability and the gene expression of key components of the gut barrier shifted sharply at the onset of solid food ingestion *in vivo*, despite milk was still predominant in the diet at that time. We found that cecal content sterile supernatant (i.e. containing a mixture of metabolites) obtained after the onset of solid food ingestion accelerated the formation of the epithelial barrier in Caco-2 cells *in vitro* and our results suggested that these effects were driven by the bacterial metabolite butyrate. Moreover, the treatment of organoids with cecal content sterile supernatant partially replicated *in vitro* the effects of solid food ingestion on the epithelial barrier *in vivo*. Altogether, our results show that the metabolites produced by the microbiota at the onset of solid food ingestion contribute to the maturation of the gut barrier at the suckling-to-weaning transition. Targeting the gut microbiota metabolic activity during this key developmental window might therefore be a promising strategy to promote intestinal homeostasis.

## Introduction

The intestinal epithelium is a physicochemical and immunological barrier against luminal antigens and enteric pathogens, yet allowing nutrients and water absorption.^1,2^ Epithelial cells strictly limit bacterial invasion through a high proliferation rate, mucus secretion, tight junction formation and innate immune responses.^3^ During the postnatal period, the maturation of the intestinal epithelium has lifelong consequences for gut and immune homeostasis.^4^ Indeed, disruption of the gut barrier in young animals increases the susceptibility to digestive and infectious diseases later in life.^5^ Understanding the mechanisms underlying the postnatal maturation of the gut barrier is therefore necessary to develop innovative strategies supporting lifelong intestinal homeostasis.

In mammals, the suckling-to-weaning dietary transition is associated with major developmental changes in the intestine, promoting the gut barrier formation.^6-10^ At the same period, the shift from maternal milk to solid food induces a profound remodeling of the gut microbiota composition, mainly characterized by an increase in diversity and a decline of facultative anaerobes.^4,11-14^ Together with genetically encoded and nutritional factors, these changes in the gut microbiota drive the intestinal ontogeny at the onset of solid food ingestion.^15,16^ For instance, experiments in germ-free mice revealed that bacterial colonization of the gut is necessary for the regulation of antimicrobial peptides expression induced by weaning.^15^ However, the mechanisms underlying the microbial control of intestinal maturation at the onset of solid food ingestion are not completely understood.

Metabolites produced by the gut bacteria are considered as key molecular intermediates between the microbiota and its host.^17^ At the suckling-to-weaning transition, there is a strong modification of the dietary substrates available for bacteria in the lumen, which is the main factor regulating the metabolic activity of the microbiota.^18^ Therefore, we hypothesized that the production of bacterial metabolites might be profoundly altered at the onset of solid food ingestion, which might constitute a signal triggering the maturation of the gut barrier.

Herein, we studied how the introduction of solid food remodeled the gut microbiota composition, its metabolic activity and whether these changes contributed to the gut barrier maturation. We used a neonatal rabbit model since it allows an accurate monitoring of suckling and early life solid food ingestion. Indeed, in this species, the dam suckles only once 5 minutes/day, allowing the separation of the pups and their dam for the rest of the day.^19^ Our results show that the alteration of the microbiota composition at the onset of solid food ingestion is associated with a major shift in the production of bacterial metabolites that coincides with the transcriptomic regulation of key components of both immune and physical gut barrier. Experiments *in vitro* on epithelial cell lines and organoids revealed that the maturation of the gut barrier at the suckling-to-weaning transition is partly induced by gut microbiota derived metabolites, notably butyrate.

## Results

### The gut microbiota composition shifts at the onset of solid food ingestion

**Figure 1.**
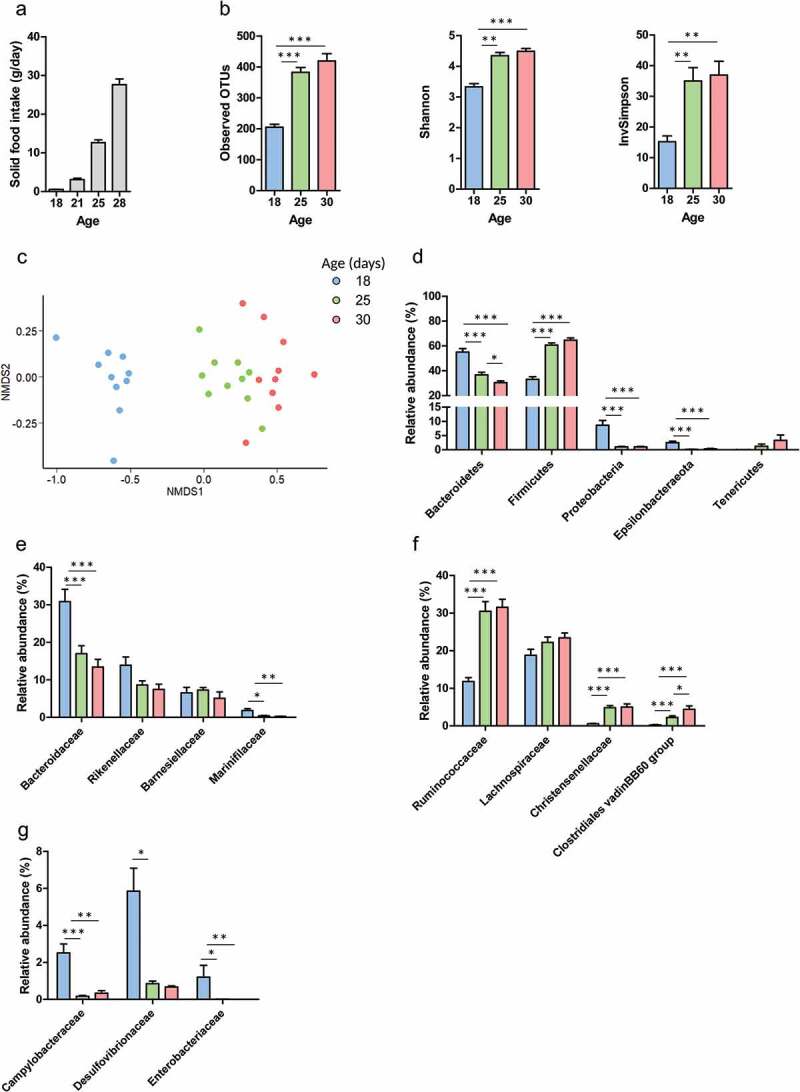
The microbiota composition shifts at the onset of solid food ingestion. (a): Solid food intake by rabbit pups per day. The microbiota composition was analyzed by 16S rRNA amplicons sequencing in cecal content of rabbits at postnatal day 18, 25 and 30. (b): α-diversity indices. (c): Non Metric Dimensional Scaling (nMDS) two-dimensional representation of the microbiota β-diversity using Bray Curtis distance calculation (stress = 11.29). (d): Relative abundance of the main phyla. (e–g): Relative abundance of families in the phylum Bacteroidetes (e), Firmicutes (f), Proteobacteria and Epsilonbacteraeota (g). Data are presented as means ± SEM, n = 10/group. Kruskal-Wallis test was used to analyze age effect, followed by pairwise Wilcoxon test to compare the mean values of each group. *: *P* < .05, **: *P* < .01, ***: *P* < .001.

We analyzed the composition of the cecum microbiota at the onset of solid food ingestion by suckling rabbits using 16S rRNA gene sequencing. We focused on three time points: postnatal day (PND) 18 (exclusively suckling), PND25 (maternal milk ingestion > solid food ingestion) and PND30 (maternal milk ingestion < solid food ingestion).^19^ As expected, solid food intake increased gradually after PND18 (Figure 1(a)). It coincided with a sharp increase in the α-diversity of the gut microbiota as indicated by the increased number of observed OTUs, Shannon and InvSimpson indices (Figure 1(b)). Additionally, β-diversity analysis using the Bray-Curtis distance revealed a strong shift in the structure of the microbiota between PND18 and 25, i.e. after the onset of solid food ingestion (Figure 1(c)). Interestingly, the modification of the microbiota structure from PND25 to 30 was much less pronounced despite solid food ingestion increased about 2-fold during this period (Figure 1(a,c)).

Bacteroidetes relative abundance gradually decreased from PND18 to 30 (Figure 1(d)). In this phylum, the relative abundance of taxa belonging to the families Bacteroidaceae (genus *Bacteroides*) and Marinifilaceae (genus *Butyricimonas*) were significantly reduced after the onset of solid food ingestion (Figure 1(e), supplemental table 1). In contrast, the relative abundance of Firmicutes reached its maximum level after PND25 (Figure 1(d)), mainly linked to an increased relative abundance of the families Ruminococcaceae (e.g. genus *Ruminococcus)*, Christensenellaceae (genus *Christensenellaceae R-7 group*) and Clostridiales vadinB660 group (Figure 1(f), supplemental table 1). The abundance of 11 out of 18 genera from the Lachnospiraceae family were also increased after the onset of solid food ingestion (supplemental table 1). Strikingly, high abundances of the phyla Proteobacteria and Epsilonbacteraeota were observed at PND18, when rabbits were exclusively suckling (Figure 1(d)). This was related to the high abundance of Desulfovibrionaceae (genus *Desulfovibrio*), Enterobacteriaceae (genus *Klebsiella*) and Campylobacteraceae (genus *Campylobacter*) (Figure 1(g), supplemental table 1). As observed for the diversity analysis, there was only few differences in the microbiota taxonomic composition between PND25 and 30 while the quantity of solid food ingested increased considerably during this time period. In summary, the introduction of solid food in the diet induced a major shift in the microbiota composition of suckling rabbits while the later increase in the quantity of solid food ingested had more limited effects.

### The metabolic activity of the gut microbiota is remodeled at the onset of solid food ingestion

**Figure 2.**
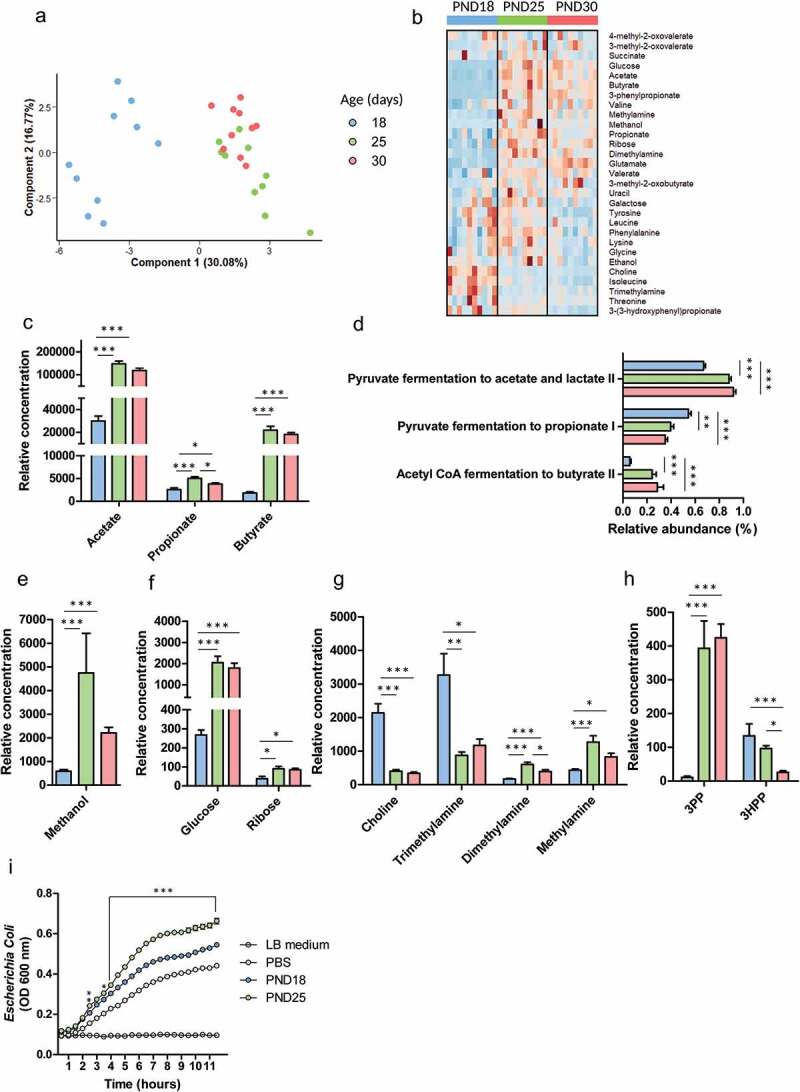
The metabolic activity of the gut microbiota is altered at the onset of solid food ingestion. The metabolome was analyzed by NMR metabolomics in cecal content of rabbits at postnatal day (PND) 18, 25 and 30. (a): Individual plot of principal component analysis (PCA). (b): Heatmap representing the relative concentration of all identified metabolites (rows) in individual samples (columns). The color represent the Z-scores (row-scaled relative concentration) from low (blue) to high values (red). Metabolites (rows) were clustered by the average method. (c): Relative concentration of the main short chain fatty acids. (d): PICRUSt2 predicted relative abundance of pathways involved in short chain fatty acids production. (e–h): Relative concentration of methanol (e), sugars (f), choline and its microbial derivatives (g), aromatic bacterial metabolites (h). 3PP:3-phenylpropionate, 3HPP: 3-(3-hydroxyphenyl)propionate. Data are presented as means ± SEM, n = 10/group. Kruskal-Wallis test was used to analyze age effect, followed by pairwise Wilcoxon test to compare the mean values of each group. (i): Growth of *Escherichia coli* in LB medium diluted 1:2 (v/v) in PBS or in a pool of sterile supernatant of cecal contents collected from rabbits at PND18 or PND25. The experiment was repeated 4 times. Data are presented as means ± SEM. Mean OD values of PND18 and PND25 were compared pairwise at each time point. *: P < .05, **: P < .01, ***: P < .001.

As a next step, we explored how solid food ingestion by suckling rabbits impacted the metabolic activity of the gut microbiota by analyzing cecal content metabolome by using NMR-based metabolomics. Principal component analysis (PCA) and heatmap representation including all the 29 identified metabolites (supplemental table 2 and supplemental Figure 1) revealed a strong modification of the cecal metabolome after the onset of solid ingestion, while only few differences were observed when the quantity of solid food ingested increased from PND25 to 30 (Figure 2(a,b)).

The concentration of short-chain fatty acids (SCFA), the main bacterial metabolites, increased after the onset of solid food ingestion (10-fold for butyrate, 5-fold for acetate and 2-fold for propionate) (Figure 2(c)). The predicted relative abundance of microbial pathways involved in acetate and butyrate production increased after PND25 while the relative abundance of the propionate production pathway was the highest at PND18 (Figure 2(d)). Moreover, we observed that solid food ingestion was associated with a high cecal concentration of methanol (Figure 2(e)), an alcohol produced by the gut microbiota notably from plant cell walls.^20^ Additionally, the cecal concentration of two sugars increased after the onset of solid food ingestion (7-fold for glucose and 2-fold for ribose) (Figure 2(f)). The drop in choline concentration at PND25 was associated with a sharp decrease in the concentration of its bacterial catabolite trimethylamine, while the concentration of further bacterial demethylation products (dimethylamine and methylamine) increased at PND25 (Figure 2(g)). The bacterial metabolite 3-phenylpropionate (3PP) was virtually absent in the cecum of exclusively suckling rabbits (PND18) while its concentration increased remarkably after PND25 (Figure 2(h)). In contrast, the concentration of the bacterial metabolite 3-(3-hydroxyphenyl)propionate (3 HPP) decreased gradually as the quantity of solid food ingested increased (Figure 2(h)). Altogether, these data indicate that the beginning of solid food ingestion by suckling rabbits remodeled the cecum metabolome, largely through a modulation of the gut microbiota metabolic activity. In the same way than observed for the microbiota composition, once solid intake was initiated, the production of bacterial metabolites was only slightly affected by the amount of solid food ingested.

Then, we tested whether the metabolome modifications observed at the onset of solid food ingestion (PND25) could modulate bacterial growth due to changes in substrate availability. We monitored the growth of *Escherichia coli* in LB medium containing PBS (negative control) or sterile supernatant of cecal content collected from rabbits at PND18 or PND25 (Figure 2(i)). Cecal content sterile supernatants from both groups stimulated the growth of *E. coli* (*P* < .001 when compared to PBS from 2 h onwards). The cecal content sterile supernatant collected at PND25 accelerated the growth of *E. coli* when compared to the cecal content sterile supernatant collected at PND18 (*P* < .001 from 4 h onwards). Thus, the modulation of luminal environment at the onset of solid food ingestion is able to promote bacterial growth.

### Epithelial barrier is altered at the onset of solid food ingestion

**Figure 3.**
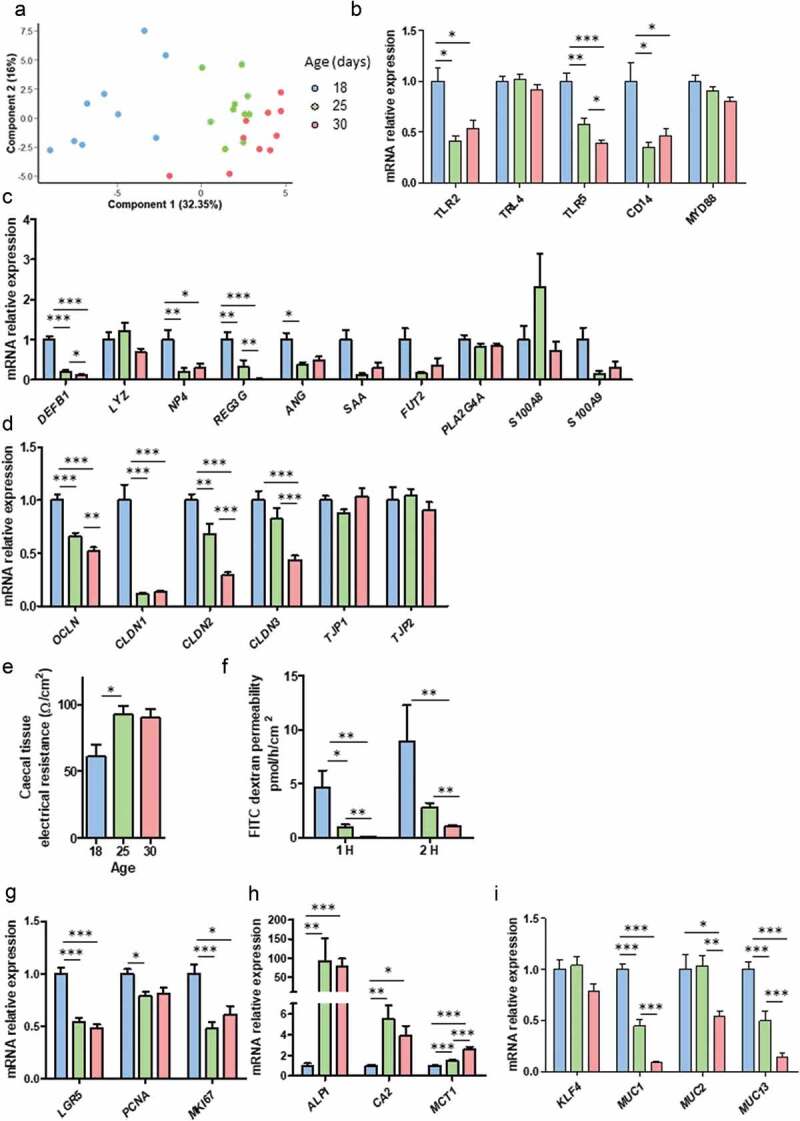
Epithelial components of the gut barrier are regulated at the onset of solid food ingestion. Gene expression was analyzed by high throughput microfluidic qPCR in the cecal mucosa of rabbits at postnatal day 18, 25 and 30. (a): Individual plot of principal component analysis (PCA). (b–d): Relative expression of genes involved in the toll-like receptors signaling pathway (b), antimicrobial defenses (c), tight junctions (d). (e): Cecal tissue electrical resistance was measured *ex vivo* in Ussing chambers. (f): Epithelial para-cellular permeability to FITC-dextran 4kDa was measured after 1 and 2 h in cecal tissue fragments mounted in Ussing chambers. (g–i): Relative expression of genes involved in epithelial proliferation (g), epithelial differentiation (h) and mucus secretion (i). Data are presented as means ± SEM, n = 9-10/group for gene expression analysis and n = 5–6 for Ussing chamber experiments. Kruskal-Wallis test was used to analyze age effect, followed by pairwise Wilcoxon test to compare the mean values of each group. *: *P* < .05, **: *P* < .01, ***: *P* < .001.

The gut microbiota is known to be a key regulator of mucosal homeostasis. Therefore, we assessed whether the modifications of the microbiota composition and metabolic activity at the onset of solid food ingestion were associated with a regulation of the cecum mucosa transcriptome, by using a high-throughput microfluidic qPCR array. PCA including all gene expressions measured indicated a strong shift in the mucosal transcriptome according to age (Figure 3(a)).

In the pattern-recognition receptor (PRR) signaling pathway, we observed a reduced gene expression of “Toll-like receptor 2” (*TLR2), TLR5* and of the TLR4 co-receptor *CD14* from PND25, i.e. after the onset of solid food ingestion (Figure 3(b)). In contrast, the gene expression of *TLR4* and the TLR adapter protein “myeloid differentiation primary response 88” (*MYD88*) remained unchanged. Among the anti-microbial peptides tested, the gene expression of “defensin β1” (*DEFB1*), “neutrophil antibiotic peptide” (*NP4*), “regenerating islet-derived protein 3 γ” (*REG3 G*) and “angiogenin” (*ANG*) were reduced from PND25 (Figure 3(c)). The expression of genes coding for the tight junction proteins “occludin” (*OCLN*), “claudin 1” (*CLDN1), CLDN2, CLDN3* were reduced from PND25, while the gene expression of “tight junction protein 1” (*TJP1*) and *TJP2* remained unchanged (Figure 3(d)). In order to translate the functional consequences for the gut barrier of these changes observed at the gene expression level, we performed *ex vivo* experiments in Ussing chambers. Cecal tissue electrical resistance increased after the onset of solid food ingestion (PND25) and this was associated with a reduction of para-cellular permeability to FITC-dextran 4kDa (Figure 3(e,f)), suggesting a reinforcement of the gut barrier.

The gene expression of the intestinal stem cells marker “leucine-rich repeat-containing G-protein coupled receptor” (*LGR5)* was reduced after the onset of solid food ingestion (Figure 3(g)). In agreement, the proliferation markers “proliferating cell nuclear antigen” (*PCNA*) and MKI67were reduced from PND25 (Figure 3(g)). Conversely, the gene expression of the absorptive epithelial cells markers “alkaline phosphatase intestinal” (*ALPI*), “carbonic anhydrase 2” (*CA2*) and “monocarboxylate transporter 1” (*MCT1*) were up-regulated after solid food ingestion (Figure 3(h)). The gene expression of “mucin 1” (*MUC1), MUC2* and *MUC3* decreased with age while the gene expression of the goblet cell differentiation factor “Krueppel-like factor 4” (*KLF4)* remained unchanged (Figure 3(i)).

**Figure 4.**
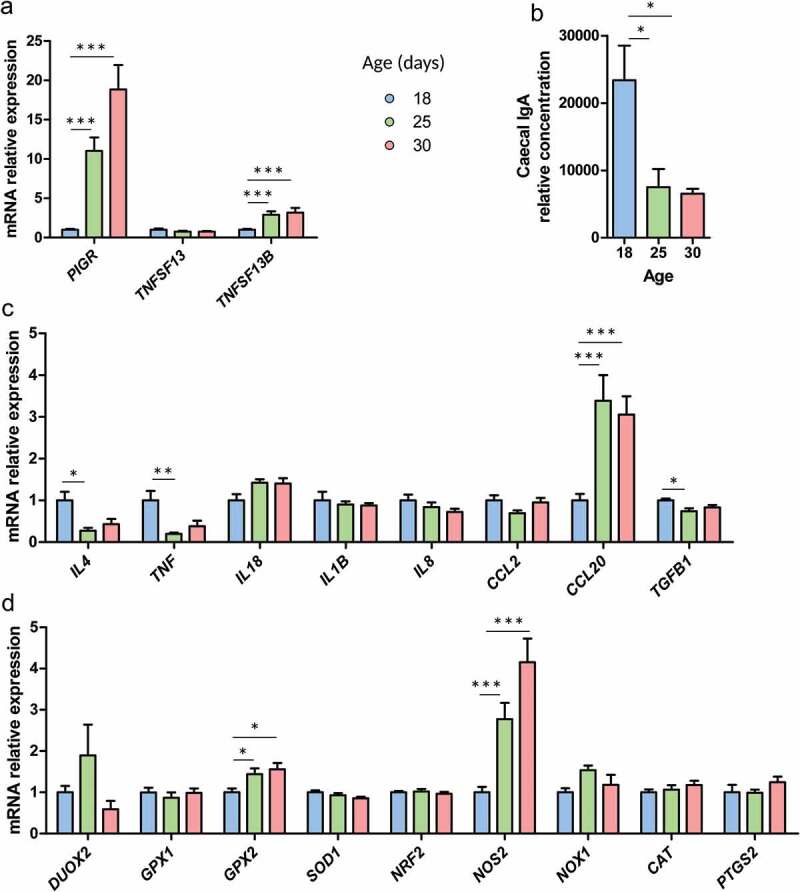
Immune and redox intestinal defenses are regulated at the onset of solid food ingestion. Gene expression was analyzed by high throughput microfluidic qPCR and immunoglobulin A (IgA) were quantified in the cecum of rabbits at postnatal day 18, 25 and 30. (a): Relative expression of genes involved in the IgA secretion pathway. (b): IgA relative concentration in the cecal content. (c–d): Relative gene expression of cytokines (c) and redox signaling proteins (d). Data are presented as relative expression means ± SEM, n = 9-10/group. Kruskal-Wallis test was used to analyze age effect, followed by pairwise Wilcoxon test to compare the mean values of each group. *: *P* < .05, **: *P* < .01, ***: *P* < .001.

Regarding the epithelial transcytosis of immunoglobulin A (IgA), the gene expression of the “polymeric immunoglobulin receptor” (*PIGR*) was more than 10-fold up-regulated after PND25 (Figure 4(a)). This was associated with an up-regulation of the survival signal for IgA-secreting plasmocyte “B cell activation factor” (*TNFSF13B*coding for the protein BAFF) while the expression of “proliferation-inducing ligand” (*TNFSF13* coding for the protein APRIL) was not changed (Figure 4(a)). Interestingly, the concentration of IgA in the cecum (both from maternal milk and endogenous origin) decreased from PND25 (Figure 4(b)). Among the cytokines tested, the gene expression of “interleukin-4” (*IL4*), “tumor necrosis factor” (*TNF*) and “transforming growth factor β1” (*TGFB1*) were down-regulated at PND25 compared to PND18 (Figure 4(c)). In contrast, the gene expression of “C-C motif chemokine ligand 20” (*CCL20*) was strongly up-regulated after the onset of solid food ingestion (Figure 4(c)). Finally, among a panel of genes involved in redox signaling, “glutathione peroxidase 2” (*GPX2*) and “nitric oxide synthase 2” (*NOS2*) were up-regulated from PND25 (Figure 4(d)). In summary, the expression of genes involved in the gut barrier function were highly regulated during the transition from maternal milk to solid food.

### Bacterial metabolites produced by the gut microbiota at the onset of solid food ingestion induce the maturation of the epithelial barrier *in vitro*

**Figure 5.**
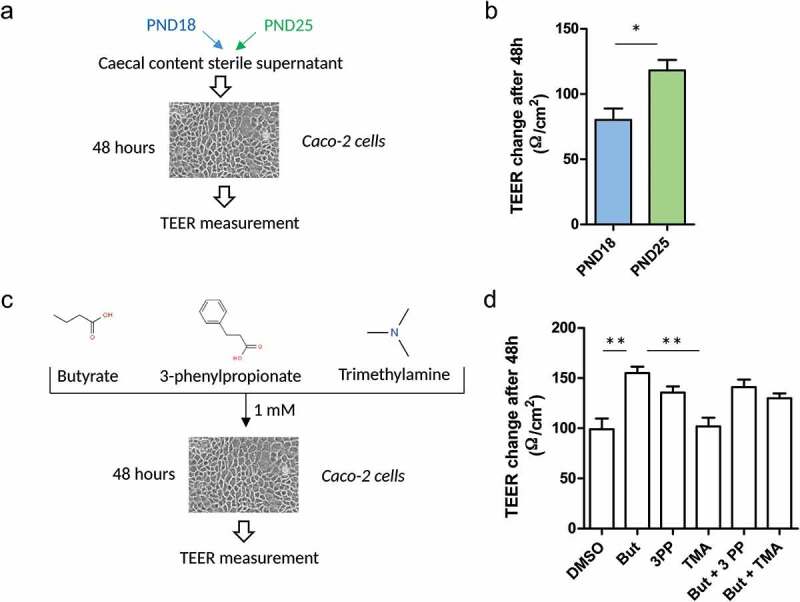
Cecum metabolites produced at the onset of solid food ingestion promote the formation of the epithelial barrier *in vitro*. (a): The epithelial cell line Caco-2 was treated for 48 hours with 10% (v/v) cecum sterile supernatant of rabbits at postnatal day (PND) 18 or 25. (b): Transepithelial electrical resistance (TEER) was measured before and after the treatment with cecal content sterile supernatants (n = 9-10/condition). (c): Caco-2 cells were treated for 48 hours with bacterial metabolites alone or in combination (1 mM). (d): TEER was measured before and after the treatment with DMSO (negative control, 0.1%), butyrate (But), 3-phenylpropionte (3PP), trimethylamine (TMA), butyrate and 3-phenylpropionte (But + 3PP), butyrate and trimethylamine (But + TMA) (n = 6-8/condition). Data are presented as means of TEER change over 48 h ± SEM. Kruskal-Wallis test was used to analyze treatment effect, followed by pairwise Wilcoxon test to compare the mean values of each group. *: *P* < .05, **: *P* < .01, ***: *P* < .001.

As a next step, we wanted to address whether the modifications of the gut microbiota induced by solid food ingestion were involved in the maturation of the gut mucosa. We hypothesized that the alteration of the production of bacterial metabolites might act as a signal regulating the epithelial barrier. Therefore, we treated confluent monolayers of Caco-2 intestinal epithelial cells with cecal content sterile supernatants (i.e. containing a mixture of luminal metabolites) prepared from rabbits exclusively suckling (PND18) or after the onset of solid food ingestion (PND25) (Figure 5(a)). After 48 hours, the PND25 cecum supernatant induced a more important increase of transepithelial electrical resistance (TEER) than the PND18 cecum supernatant (Figure 5(b)). These results suggest that the metabolites present in the cecum after solid food ingestion are more prone to induce the development of the epithelial barrier when compared to the metabolites present in the cecum of exclusively suckling rabbits.

In order to identify which compounds present in the cecum supernatant were responsible for these effects, we treated Caco-2 cells confluent monolayers with bacterial metabolites whose concentration increased (butyrate and 3-phenylpropiontate, Figure 2(c,h)) or decreased (trimethylamine, Figure 2(g)) after solid food ingestion (Figure 5(c)). Butyrate induced a significantly more important TEER increase than DMSO (control) or trimethylamine (Figure 5(d)). 3-phenylpropionate and the combination of butyrate + 3-phenylpropionate or butyrate + trimethylamine induced a more important TEER increase than DMSO or trimethylamine, but these effects were not statistically significant (Figure 5(d)). Taken together, these *in vitro* experiments on Caco-2 cells show that the metabolites present in the cecum after solid food ingestion promote the formation of the epithelial barrier, and that the bacterial metabolite butyrate was able to reproduce this effect.

### Alteration of the luminal environment at the onset of solid food ingestion regulates gene expression in cecum organoids

**Figure 6.**
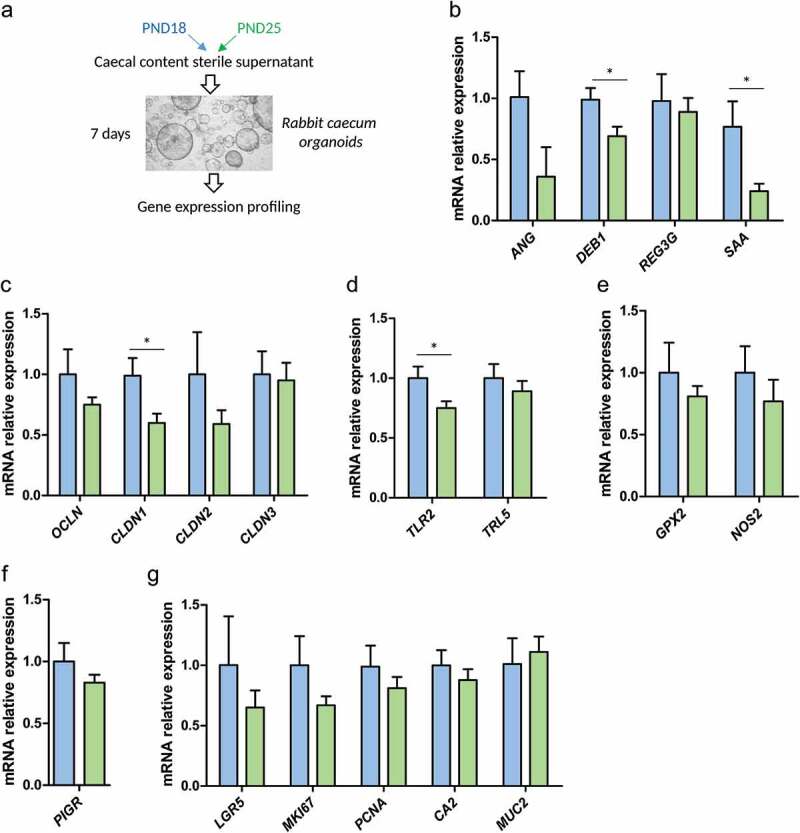
Cecum metabolites produced at the onset of solid regulate epithelial gene expression in organoids. (a) – Gene expression was analyzed by high throughput microfluidic qPCR in rabbit cecum organoids treated for 7 days with 10% (v/v) cecal content sterile supernatant of rabbits at postnatal day (PND) 18 or 25. (b–g): relative expression of genes involved in antimicrobial defenses (b), tight junctions (c), toll-like receptors signaling pathway (d), redox signaling (e), immunoglobulin A secretion (f) and epithelial cell proliferation and differentiation (g). Data are presented as means ± SEM, n = 9-10/group. Kruskal-Wallis test was used to analyze treatment effect. *: *P* < .05, **: *P* < .01, ***: *P* < .001.

Since bacterial metabolites produced at the onset of solid food ingestion are able to accelerate the epithelial barrier formation *in vitro*, we next assessed whether they were involved in the regulation of gene expression that we observed *in vivo*. We treated rabbit cecum organoids for 7 days with cecal content sterile supernatant prepared from rabbits exclusively suckling (PND18) or after the onset of solid food ingestion (PND25) (Figure 6(a)). Interestingly, the treatment of organoids with cecum supernatants was able to reproduce some of the modifications of gene expression induced by the onset of solid food ingestion *in vivo*. Indeed, when compared to PND18 cecum supernatant, PND25 cecum supernatant induced a lower gene expression of *ANG* (*p* = .06), *DEFB1, CLDN1* and *TLR2* (Figure 6(b-d)), these genes were also down-regulated after the onset of solid food ingestion *in vivo* (Figure 3(b–d)). PND25 cecum supernatant also reduced the expression of “serum amyloid A” (*SAA)* (Figure 6(b)), this trend was also observed *in vivo* at PND25 (Figure 3(c)). However, some of the modifications of gene expression induced *in vivo* by the onset of solid food ingestion were not replicated in organoids. For instance, the cecum sterile supernatant did not alter the expression of *REG3G, TLR5, NOS2* or *PIGR* (Figure 6), suggesting that other factors than those presents in the metabolite mixture were necessary to regulate these genes at the onset of solid food ingestion. Importantly, the overall organoid homeostasis was not altered by the cecum supernatants as shown by the unchanged expression of stem cell, proliferation and differentiation markers (Figure 6(g)). In summary, these *in vitro* experiments in organoids show that the alteration of the luminal environment (i.e. metabolites) induced by the introduction of solid food regulates gene expression in epithelial cells. Some of these transcriptomic alterations were reminiscent of the in *vivo* observations, suggesting that cecum metabolites contribute to the regulation of the gut barrier at the onset of solid food ingestion.

## Discussion

Maternal milk shapes the neonatal microbiota by providing fat, protein, oligosaccharides and immunological factors such as immunoglobulins and antimicrobial peptides.^21^ The high abundance of *Bacteroides* (the most abundant genus in Bacteroidetes) in exclusively suckling rabbits is a typical feature of a “milk-oriented microbiota”.^22-24^ Indeed, members of this genus have the capacity to degrade milk oligosaccharides externally.^22^ It has been proposed that a possible consequence of this metabolic activity is the luminal release of monosaccharides that might promote the growth of potentially pathogenic bacteria such as *Enterobacteriacae* species through cross-feeding reactions.^22^ Indeed, the cecum of exclusively suckling rabbits was characterized by a relatively high abundance of Proteobacteria *(Enterobacteriaceae, Desulfovibrionaceae*) and Epsilonbacteraeota (*Campylobacter)*, which encompass potential pathobionts. Moreover, bacteria in these taxa are frequently facultative anaerobes, making them particularly adapted to the oxygen present in the neonatal gut.^25^ Interestingly, we also found that the cecal content of exclusively suckling rabbits was characterized by a high concentration of choline, an essential nutrient that plays a key role in neonatal development.^26^ Since mammalian milk is rich in choline, it suggests an incomplete absorption of this nutrient, as previously observed when high amounts are consumed.^27^ This was associated with a high cecal concentration of trimethylamine, a metabolite produced by the gut microbiota notably through choline degradation.^28^ Interestingly, genera in the families Desulfovibrionaceae *(Desulfovibrio)* and Enterobacteriaceae *(Klebsiella)* that were abundant in exclusively suckling rabbits encompass members that are able to degrade choline into trimethylamine.^29^ Altogether, our results obtained at PND18 suggest that maternal milk provides dietary substrates driving both the composition and the metabolic activity of the gut microbiota.

The suckling-to-weaning transition profoundly alters the substrates available for gut bacteria since plant-based solid foods are rich in complex carbohydrates such as starch and fibers. The introduction of solid food increased the diversity of the microbiota and reshaped its structure, as observed in previous studies.^11,23,30,31^ Strictly anaerobic bacteria able to degrade complex polysaccharides, such as members of the family Ruminococcaceae, became predominant in the digestive ecosystem, due to their capacity to use the newly available plant-derived substrates.^32^ This was accompanied by an important increase in cecal concentration of sugars (glucose and ribose) and microbial metabolites derived from carbohydrates catabolism, such as SCFA and methanol.^17^ Moreover, the concentration of the gut microbiota derived 3-phenylpropionate increased considerably after the onset of solid food ingestion, probably due to the bacterial degradation of plant polyphenols.^33^ The more rapid growth of *E. coli* when it was incubated *in vitro* with the sterile supernatant of cecal contents at PND25 compared to PND18 is consistent with the increased availability of bacterial growth substrates (e.g. SCFA or glucose) present in the cecum after the onset of solid food ingestion. Strikingly, the major shift in the microbiota composition and metabolic activity observed at the onset of solid food ingestion occurred while milk intake was still predominant^19^ and these effects were not much amplified when the amount of solid food ingested increased. Thus, our results suggest that the introduction of limited amounts of plant-derived substrates is sufficient to overwhelm the influence of milk on the microbiota.

Importantly, the modulation of the microbiota composition and metabolic activity observed at the suckling-to-weaning transition was associated with the maturation of the gut mucosa at the transcriptional level. Epithelial cells proportions shifted after the onset of solid food ingestion, as shown by the increased expression of absorptive cells markers (*ALPI, CA2, MCT1*) and a reduction of markers of stem (*LGR5*), proliferating (*MKI67, PCNA*) and secretory goblet cells (*MUC2*).^34^ This differentiation of epithelial cells toward an absorptive phenotype was associated with the regulation of the expression of genes involved in the intestinal barrier in the cecum mucosa of rabbits after the onset of solid food ingestion, as previously observed in mice models.^6,8^ In rabbit cecum, the gene expression of tight junction proteins *OCLN, CLDN1, CLDN2* and *CLDN3* was reduced at the onset of solid food ingestion while *TJP1* and *TJP2* remained unchanged. Our results are in agreement with previous reports showing a gradual reduction of *CLDN1* and *CLDN2* expression during the postnatal period in mice jejunum.^6,35^ However, in contrast with our results, *CLDN3* was found to be up-regulated after weaning in mice jejunum and ileum,^35,36^ probably because of intestinal segments or species particularities. The downregulation of tight junction protein gene expression at the onset of solid food ingestion was associated with an increased electrical resistance of the cecal tissue and with a decreased *ex vivo* para-cellular permeability to FITC-dextran. This improvement of the barrier function at the suckling to weaning transition is consistent with previous studies in rabbits and mice.^36,37^ The link between the reduction of tight junction protein gene expression and the reduction of para-cellular permeability is difficult to interpret since claudins that we found regulated are either pore-forming (e.g. claudin 2) or sealing (e.g. claudin 1 and 3) and the role of occludin in paracellular barrier function is controversial.^38,39^ Moreover, tight junction proteins follow a different expression pattern according to epithelial cell types (e.g. stem cells or absorptive cells).^40^ Thus, the regulation of tight junction protein gene expression observed at the onset of solid food ingestion might also be linked to a modification of the proportion of the different epithelial cell types, as suggested by the gene expression of the stem cell marker *LGR5* or absorptive cell marker *ALPI* that were respectively down and upregulated after PND25.

The TLR signaling pathway was previously shown to be a key regulator of gene expression in the intestine at the suckling-to-weaning transition.^6^ In rabbit cecum mucosa, we observed a down-regulation of *TLR2* and *TLR5* expression after the onset of solid food ingestion. A similar down-regulation of *TLR5* expression after weaning was previously observed in mice intestinal epithelial cells.^6-8^ A recent study demonstrated that the high neonatal *TLR5* expression in the epithelium plays a key role in the selection of bacteria colonizing the intestine in early life, notably through the induction of *REG3γ.*^7^ This antimicrobial peptide and others secreted by epithelial cells are essential components of the innate immune defenses of the intestine.^41,42^ In rabbit cecum mucosa, the expression of several antimicrobial peptides was strongly reduced after the onset of solid food ingestion (*REG3G, ANG, DEFB1, NP4)* while some others remained unchanged (e.g. *LYZ, PLA2G4A*). The expression of antimicrobial peptides was previously shown to be highly regulated in the developing intestine.^6,8,15,43^ In contrast to our results, most of these studies reported an up-regulation of antimicrobial peptides expression after weaning in mice. However, it is worth noting that these studies were conducted in the small intestine, large differences in antimicrobial expression patterns being observed according to the intestinal segment.^44^ Moreover, complex regulations of antimicrobial peptides at the suckling-to-weaning transition were reported, some genes being up-regulated while other being down-regulated, even within the same family.^6^ We hypothesize that the down-regulation of antimicrobial peptides expression in the rabbits cecum mucosa after the onset of solid food ingestion can be compensated by several mechanisms including the strong up-regulation of *CCL20*, a chemokine with antibacterial activity,^41^ or through the up-regulation of *NOS2*, coding for the inducible nitric oxide (NO) synthase producing antimicrobial NO.^45^ Additionally, genes involved in the survival of IgA-secreting plasmocytes (*TNFSF13B*) and epithelial transcytosis of IgA (*PIGR*) were also strongly induced at the introduction of solid food, as described previously in mice models.^6,8,46^ This process corresponds to the development of the pup intestinal adaptive immune system that compensates for the gradual decreased supply of immunoglobulins from maternal milk (i.e. passive immunity).^46^

Collectively, our results highlight the coordination of microbiota and gut barrier maturation at the onset of solid food ingestion. Since previous studies showed that the colonization of the gut by the microbiota is directly involved in the maturation of the intestinal epithelium at the suckling-to-weaning transition,^15^ we explored how gut bacteria contribute to this developmental process. The use of cecum sterile supernatant allowed us to investigate the role played by metabolites independently of the presence of bacteria. Strikingly, the mixture of metabolites present in the cecum after the onset of solid food ingestion was sufficient to promote the formation of the epithelial barrier in the Caco-2 cell line, consistent with our data obtained *ex vivo* in Ussing chambers. Our results suggest that this effect was mainly linked to the increased concentration of butyrate, observed when plant-derived substrates were ingested. This result is in agreement with previous reports showing that butyrate strengthens the epithelial barrier.^47,48^ However, we cannot exclude that other compounds present in the sterile supernatant of cecal content (e.g. endotoxins) might have contributed to their effects on epithelial barrier formation.

In addition, our experiments in organoids indicated that the mixture of metabolites present in the cecum supernatant at the onset of solid food ingestion was able to mimic some of the transcriptomic regulations observed *in vivo* (*ANG, DEFB1, TLR2, CLDN1)*. These results suggest that metabolites produced by the microbiota after the onset of solid food ingestion directly contribute to the maturation of the epithelial barrier at the suckling-to-weaning transition. Butyrate might play a preponderant role in this process since it was for instance shown to reduce the expression of some claudins.^48,49^ However, more studies are needed to decipher the role played by each metabolite, alone or in combination with others. Some of the developmental regulation observed *in vivo* were not reproduced in organoids (e.g. epithelial differentiation, up-regulation of the IgA secretion pathway), suggesting either that they require the presence of bacteria or that they are genetically encoded.

Regarding the potential mode of action of bacterial metabolites on epithelial cells, we hypothesize that it might involve epigenetic modifications. Indeed, the epigenetic mechanisms orchestrating the postnatal development of the intestinal epithelium are partly guided by the gut microbiota.^8,50^ Interestingly, several metabolites which concentration was altered at the onset of solid food ingestion have been shown to be involved in epigenetic regulation. In exclusively suckling rabbits, the intense bacterial conversion of choline to trimethylamine might reduce the availability of methyl donors for the epithelial cells and consequently modify DNA or histone methylation patterns.^51^ Moreover, the increased butyrate concentration at the onset of solid food ingestion might regulate gene expression in epithelial cells through its capacity to inhibit histone deacetylase.^52^ Bacterial derivatives of phenolic acids, such as 3-phenylpropionate that was produced when plant-derived substrates were available, were also shown to inhibit histone deacetylase.^52^ Further experiments are needed to decipher these potential links between early life nutrition, bacterial metabolites and epigenetic control of intestinal development.

In conclusion, our results show that the introduction of solid food induces the co-maturation of the gut microbiota and of the gut mucosa. We provide new data indicating that the shift in bacterial metabolism at the suckling-to-weaning transition is directly involved in the development of the epithelial barrier. Targeting the production of bacterial metabolites in early life might therefore be a promising strategy to promote the postnatal development of the intestine and thereby guarantee gut homeostasis and health.

## Material and methods

### Animal experiments

The experiment was carried out at the PECTOUL Experimental Unit (INRAE, Castanet-Tolosan, France). Animals were handled according to the European Union recommendations on the protection of animals used for scientific purpose (2010/63/EU) and in agreement with French legislation (NOR:AGRG1238753A 2013). Animal experiments received the approval of the local ethical committee (SSA_2018_010 and SSA_2019_001). The rabbits studied were the terminal crossbreed from two commercial lines (maternal line: Hyplus PS19, paternal line: Hyplus PS59; Hypharm, France). Dams were multiparous with an average parity rank of five. Dams (n = 10) were housed individually in a wire cage (61 x 69 × 49 cm) equipped with a closed nest for the pups (39 x 27 × 35 cm). The litter size was standardized to ten pups by cross-fostering or culling at PND3. Rabbit pups were suckled daily from birth to PND30. Commercial pellets (StabiPro, Terrya, Rignac, France) were provided *ad libitum* to the pups from PND15 (the chemical composition of the diet is shown in supplemental table 3). The food ingestion of the litter was monitored separately from the dam. At PND18, PND25 and PND30, ten male or female rabbits (1 pup/litter) were slaughtered by electronarcosis and exsanguination. The cecum was isolated and the cecal content was collected. The cecal tissue was collected, washed in ice-cold PBS and snap-frozen in liquid nitrogen. All samples were stored at −80°C. For *ex vivo* intestinal permeability assay, the animal experiment was repeated with 6 litters and the cecal tissue was collected at PND18, PND25 and PND30 (n = 6 pups/group, 1pup/litter) and stored in cold PBS until mounting in Ussing chambers.

### Caco-2 cells culture

The human intestinal epithelial cell line Caco-2 (passages 50–56) was maintained in T75 flasks at 37°C under 5% CO_2_ atmosphere in DMEM high glucose GlutaMAX^TM^ Supplement pyruvate (Thermo Fisher Scientific, Waltham, MA) with 10% fetal bovine serum (Thermo Fisher Scientific) and 1% penicillin/streptomycin (Sigma-Aldrich, St-Louis, MO). The medium was replaced every 2–3 days and the cells were weekly subcultured by partial digestion with EDTA-trypsin 0.25% w/v (Thermo Fisher Scientific). Membrane inserts (PET insert, pores Ø 0.45 µM, Corning, NY) were seeded with 4.10^4^ cells/well in 24-well plates until confluency was reached (about 15 days later), as evaluated by the stabilization of TEER measured with Millicell ERS-2 Volt-Ohm Meter (Merck Millipore, Burlington, MA). The high TEER mean value (484 Ω/cm^2^) indicated that Caco-2 cells were differentiated.^53^ Monolayers were treated for 48 hours at the apical side with (i) the sterile supernatant of cecal content diluted in cell culture media (10% v/v) or with (ii) bacterial metabolites alone or in combination (1 mM). TEER was measured before and after the treatments. Cecal content sterile supernatants were prepared by homogenizing cecal content in PBS (10% w/v) before centrifugation (12 000 g, 10 min, 4°C) and filtration (0.22 µM) in an aseptic environment. Stock solution of bacterial metabolites (butyrate, trimethylamine and 3-phenylpropionate, Sigma-Aldrich) were prepared in DMSO and filtered (0.22 µM). DMSO was used as a negative control at a final concentration of 0.1%.

### Organoids culture

Cecum epithelial crypts were isolated from a 30-day-old male rabbit by incubation of cecal mucosa in a dissociation solution (9 mM EDTA [Thermo Fisher Scientific], 3 mM DTT [Sigma-Aldrich], 10 µM Y27632 [ATCC, Manassas, VA] and 1% penicillin/streptomycin [Sigma-Aldrich]) for 30 minutes under agitation. The mucosa was transferred in PBS (5 mL) and crypts were detached by manual shaking (1 min). After centrifugation (500 rpm, 5 min, 4°C), the crypts were counted and resuspended in ice-cold matrigel (Corning, NY) and seeded in pre-warmed 48-well plates at a density of 150 crypts/25 µL of matrigel. After polymerization (37°C, 20 min), 250 µL organoid growth medium was added to each well. The organoid growth medium (modified from a previous publication^54^) was prepared in DMEM high glucose GlutaMAX^TM^ Supplement pyruvate (Thermo Fisher Scientific) with 10% fetal bovine serum (Thermo Fisher Scientific), 1% penicillin/streptomycin (Sigma-Aldrich), 1 mM HEPES (Sigma-Aldrich), 0.5 mM N-acetyl cysteine (Sigma-Aldrich), 10 µM SB431542 (Sigma-Aldrich), 10 µM CHIR99021 (Sigma-Aldrich), 10 µM Y27632 (ATCC) and 0.2 µM LDN193189 (Sigma-Aldrich). The organoid culture medium was replaced every 2–3 days. Every week, organoids were broken by pipetting before centrifugation (500 rpm, 5 min, 4°C) of the cell aggregates and re-seeded in matrigel with a dilution ratio 1:8. Organoids (passage 2) were treated for 7 days with cecum sterile supernatant (prepared as described above) diluted (1:10 v/v) in the culture medium. The media containing the cecum supernatant was replaced every 2–3 days.

### Escherichia coli *growth measurement*

Three colonies of *Escherichia coli* K-12 MG1665 grown in LB agar plates were suspended in 5 mL of LB liquid medium. *E. coli* suspension was diluted 1:2 (v/v) in sterile PBS or in a pool of cecal content sterile supernatants prepared as described above. *E. coli* growth in 96-wells plates (100 µL/well) was monitored by measurement of absorbance at 600 nm with GloMax® Discover plate reader (Promega, Madison, WI) every 30 min during 12 h at 37°C under stirring.

### 16S rRNA gene sequencing and sequences analysis

Cecal content DNA was extracted using Quick-DNA Fecal/Soil Microbe 96 Kit (ZymoResearch, Irvine, CA) and the 16S rRNA V3-V4 region was amplified by PCR and sequenced by MiSeq Illumina Sequencing as previously described.^55^ Sequencing reads were deposited in the National Center for Biotechnology Information Sequence Read Archive (SRA accession: PRJNA572565). 16S rDNA amplicon sequences were analyzed using the FROGS pipeline according to standard operating procedures.^56^ Amplicons were filtered according to their size (350–500 nucleotides) and clustered into OTUs using Swarm (aggregation distance:d=1 + d=3). After chimera removal, OTUs were kept when present in at least 3 samples or representing more than 0.005% of the total number of sequences. OTUs affiliation was performed using the reference database silva132 16 S with a minimum pintail quality of 80.^57^ The mean number of reads per sample was 18 652 (min: 13 642 – max: 28 612). The functional potential of the microbiota was predicted by using PICRUSt2^58^ according to the guidelines with the unrarefied OTU abundance table as input. Relative predicted abundance of MetaCyc pathways were calculated by dividing the abundance of each pathway by the sum of all pathway abundances per sample.

### NMR metabolomics

Cecal contents (100 mg) were homogenized in 500 µL phosphate buffer (prepared in D_2_O, pH7, TSP 1 mM) in 2 mL FastPrep tubes (Lysing D matrix) by using a FastPrep Instrument (MP biomedicals, Irvine, CA). After centrifugation (12 000 g, 4°C, 10 min), supernatants were collected. The extraction step was repeated on the pellet. Supernatants were pooled and centrifuged twice (18 000 g, 30 min, 4°C). The resulting supernatant (600 µL) was transferred to a 5 mm NMR tube. All NMR spectra were obtained with an Avance III HD NMR spectrometer operating at 600.13 MHz for ^1^H resonance frequency using a 5 mm inverse detection CryoProbe (Bruker Biospin, Rheinstetten, Germany) in the MetaboHUB-MetaToul-AXIOM metabolomics platform (Toulouse, France). ^1^H NMR spectra were acquired at 300 K using the Carr-Purcell-Meiboom-Gill spin-echo pulse sequence with presaturation. Pre-processing of the spectra (group delay correction, solvent suppression, apodization with a line broadening of 0.3 Hz, Fourier transform, zero order phase correction, shift referencing on TSP, baseline correction, setting of negative values to zero) was performed in the Galaxy tool Workflow4Metabolomics following guidelines.^59^ After water region (4.5–5.1 ppm) exclusion, spectra (0.5–9 ppm) were bucketed (0.01 ppm bucket width) and normalized by sample weight in Workflow4Metabolomics. Representative samples were characterized by 2D NMR experiments (^1^H-^1^H COSY and ^13^C-^1^H HSQC). For metabolite identification, 1D and 2D NMR spectra of pure compounds prepared in the same buffer and acquired with the same spectrometer were overlayed with sample spectra. Annotated representative spectra are presented in supplemental Figure 1. For each identified metabolite, buckets non-overlapping with other metabolites were selected for the quantification (supplemental table 2).

### Gene expression profiling

Cecal tissues were homogenized in TRI reagent (ZymoResearch, Irvine, CA) with one sterile stainless steel 5 mm diameter bead (Qiagen) by using a TissueLyzer (Qiagen) with two 3 min cycles at 30 Hz. Organoids (pool of 4 wells) were washed in PBS and homogenized by vigorous pipetting in TRI reagent. After centrifugation (12 000 g, 4°C, 10 min), 300 µL of supernatant was used for RNA extraction by using Direct-zol kit (ZymoResearch) following the manufacturer instruction, including a DNAse I treatment. RNA concentration and quality were analyzed with NanoDrop 8000 (Thermo Fisher Scientific). cDNA were prepared from 1 µg RNA with Superscript II Reverse Transcriptase (ThermoFisher Scientific) following the manufacturer’s instructions. High throughput real-time qPCR was performed using the Biomark microfluidic system using a 96.96 Dynamic Array™ IFC for gene expression (Fluidigm, San Francisco, CA) according to the manufacturer’s recommendations. The sequences of the primers used are presented in supplemental table 4. Data were analyzed with the 2^−ΔΔCt^ method with *GAPDH* gene expression used as a reference.^60^

### Immunoglobulin A quantification

Cecal content was diluted at 50 mg/mL in TBS buffer. After shaking thoroughly, the samples were centrifuged (3000 g, 10 min, 4°C). The supernatants were collected and stored at −20°C until analysis. The total cecal IgA contents were determined in duplicates by sandwich ELISA in 96-well plates coated with specific polyclonal goat anti-rabbit IgA antibody (Bethyl Laboratories, Montgomery, TX). Ten samples were pooled to build a reference sample for the standard curve elaboration. Appropriate sample dilutions were performed according to the rabbit age (from 1:12 to 1:800). Plates were read at 450 nm in GloMax® Discover plate reader (Promega, Madison, WI). Standard curve were based on a four-parametric model. Relative IgA concentrations were then interpolated from this curve and samples with intra-assay coefficients of variation lower than 20% were selected.

### Intestinal permeability measurement in Ussing chambers

Cecal tissue fragments were mounted in Ussing chambers (Physiologic Instruments, San Diego, CA) exposing a surface area measuring 0.1 cm^2^. Tissues were bathed for 2 h in oxygenated thermostated Kreb’s solution (Sigma-Aldrich). Electrical resistance was recorded at regular interval and the mean value over the 2 h period of experiment was calculated. Fluorescein Isothiocyanate (FITC)-Dextran 4kDa 2.2 mg/mL was added to the mucosal compartment. Epithelial para-cellular permeability to FITC-dextran was determined by measuring the fluorescence intensity in the serosal compartment after 1 and 2 h at 485 nm/525 nm using an automatic Infinite M200 microplate reader (Tecan, Männedorf, Switzerland).

### Statistical analysis

All statistical analyses were performed using the R software (version 3.5.1). The microbiota composition analysis was performed using the Phyloseq package.^61^ For α and β diversity analyses, the samples were rarefied to even sequencing depth (13 642 reads per sample) using the function *rarefy_even_depth*. Observed OTUs, Shannon and InvSimpson α-diversity indices were calculated using the function *estimate_richness*. The β-diversity was analyzed using the Bray-Curtis distance with the function *ordinate* and plotted by non-Metric Dimensional Scaling (nMDS) using the *plot_ordination* function. Taxa differential abundance analysis was performed with unrarefied data. OTUs representing less than 0.05% of the total number of sequences were filtered out using the function *prune_taxa*. OTUs were agglomerated at phylum, family or genus level with the function *tax_glom*. Relative abundances were calculated at each taxonomic level using the function *transform_sample_counts*.

PCA was performed with the function *pca* in the mixOmics package.^62^ The heatmap was created using the function *heatplot* in the made4 package. All univariate analyses^63^ were performed with a non-parametric Kruskal-Wallis test using the function *kruskal.test. P*-values were adjusted for multiple tests with the false discovery rate method (*p.adjust* function) for microbiome and metabolome data. Mean values of each group were compared pairwise with a Wilcoxon test using the function *pairwise.wilcox.test* and holm correction.

## Supplementary Material

Supplemental Material
