# Supplementary material for: Gut microbiota derived metabolites contribute to intestinal barrier maturation at the suckling-to-weaning transition
Source: Gut Microbes. 2020 Apr 30;11(5):1268–86. doi: 10.1080/19490976.2020.1747335 (PMC7524271; doi:10.1080/19490976.2020.1747335)
Supplement: Supplemental Material [file KGMI_A_1747335_SM6366.zip › Supplementary information/SUPPLEMENTAL TABLES.docx]

**SUPPLEMENTAL TABLES**

Supplemental table 1 (Excel file): **Relative abundances of bacterial genera.** The microbiota composition was analyzed by 16S rRNA amplicons sequencing in caecal content of rabbits at postnatal day 18, 25 and 30. The mean relative abundance of identified genera are presented at each age (n=10/group). Kruskal-Wallis test was used to analyze age effect, followed by pairwise Wilcoxon test to compare the mean values of each group. *:P<0.05, **:P<0.01, ***P<0.001.

Supplemental table 2: **Identification of metabolites in caecal content NMR spectra.** The numbers are reported in representative spectra in supplemental figure 1. s: singulet, d: doublet, t: triplet, q: quintuplet; m: multiplet, *: indicate the peak used for quantification.

|  | Metabolite | δ^1^H (ppm) |
| --- | --- | --- |
| 1 | Butyrate | 0.90* (t), 1.56 (m), 2.16 (t) |
| 2 | 4-methyl-2-oxovalerate | 0.94 (d), 2.62* (d) |
| 3 | Isoleucine | 0.94 (t), 1.01* (d) |
| 4 | Leucine | 0.97* (t) |
| 5 | Valine | 1.00* (d), 1.05 (d) |
| 6 | Propionate | 1.06* (t), 2.19 (q) |
| 7 | 3-methyl-2-oxovalerate | 1.10* (d) |
| 8 | 3-methyl-2-oxobutyrate | 1.13* (d) |
| 9 | Ethanol | 1.18* (t) |
| 10 | Valerate | 0.89 (t), 1.30* (m), 1.53 (m), 2.19 (t) |
| 11 | Threonine | 1.34* (d), 3.60 (d), 4.26 (m) |
| 12 | Lysine | 1.74* (m), 3.03 (t), 3.77 (t) |
| 13 | Acetate | 1.92 *(s) |
| 14 | Glutamate | 2.08 (m), 2.35* (m) |
| 15 | Succinate | 2.41* (s) |
| 16 | 3-(3-hydroxyphenyl)propionate | 2.48 (t), 2.85 (t), 6.76 (d), 6.80* (s), 6.87 (d), 7.25 (t) |
| 17 | Methylamine | 2.60* (s) |
| 18 | Dimethylamine | 2.72* (s) |
| 19 | Trimethylamine | 2.89* (s) |
| 20 | 3-phenylpropionate | 2.50 (t), 2.90* (t), 7.27 (t), 7.32 (d), 7.37 (t) |
| 21 | Choline | 3.21* (s) |
| 22 | Glucose | 3.25 (t), 3.40 – 3.56 (m), 3.71 – 3.91 (m), 5.24* (d) |
| 23 | Methanol | 3.36* (s) |
| 24 | Glycine | 3.57* (s) |
| 25 | Ribose | 5.26 (s), 5.39* (d) |
| 26 | Galactose | 5.27* (d) |
| 27 | Uracil | 5.81* (d), 7.55 (d) |
| 28 | Tyrosine | 6.91 (d), 7.20* (d) |
| 29 | Phenyalanine | 7.33 (d), 7.38 (t), 7.43* (t) |

Supplemental table 3: **Chemical composition of the diet.**

|  | Chemical composition (g/kg) |
| --- | --- |
| Crude protein | 176 |
| Crude fat | 25 |
| Crude cellulose | 114 |
| Acid detergent Lignin | 51 |
| Acid detergent fiber | 165 |
| Neutral detergent fiber | 319 |
| Ash | 64 |
| Starch | 131 |
| Sugars^1^ | 64 |
| Digestible fiber^1^ | 213 |
| Digestible protein^1^ | 125 |
| Digestible energy (MJ/kg) ^1^ | 9.9 |

^1^: Caculated with WUFFDA software according to tables of ingredients ^63^

Supplemental table 4: **qPCR primer sequences.**

| Gene symbol | Forward (5’- 3’) | Reverse (5’- 3’) |
| --- | --- | --- |
| *ALPI* | TGCTCTCGGTGATGTACAGG | TCACGTCGATGTCCATGTTG |
| *ANG* | CTTTCCAGGTCACCACTTGC | TTTTGGCAGCACACACTCAA |
| *CA2* | TGGGGCTCATCCGATGGACA | GCCTGGTGTAGCACTGCCAA |
| *CAT* | TCCCGATACTCACCGTCATC | CGTTCAGCACTTTCACGTAGA |
| *CCL2* | TTCACCAACAAGACCATCTCA | TGGGTTGTGGAATAAGAGGTCA |
| *CCL20* | GTGTGCAGATCCGAAGAAGG | GCTAGACCTTCAATGATGTGCA |
| *CD14* | TAGACCTCAGCCACAACTCG | AGGTTCCAGACATCCCCATC |
| *CLDN1* | TCCAGTGCAAAGTCTTCGAC | CAGCCAGTGAAAAGAGCCTG |
| *CLDN2* | CATCGTGACAGCAGTTGGTT | CCTGAGCAGCCTGGATATCA |
| *CLDN3* | CATGTCTATGGGCCTGGAGA | CTGCACCACACAGTTCATCC |
| *DEFB1* | TGACCATTACAAATGCGCCA | CGTAGCAGGTACCCTCGATT |
| *DUOX2* | ACGCGGATGGCGTGTATCAA | CTCACCACGTCGGAGAGCAC |
| *FUT2* | TCATGCCTCAGGTGTGGAAG | TGGTCATGACGGTGTGGTTA |
| *GAPDH* | AGGTCGGAGTGAACGGATT | ATGGCGACAACATCCACTTT |
| *GPX1* | ATTGAGAATGTGGCGTCGC | CATGAAGTTGGGCTCGAACC |
| *GPX2* | GTAGACTTCAACACGTTCCGG | TTGAGGTAGGTGAAGACGGG |
| *IL18* | TGTAAGCCTCTCTGTGAAGTGT | TCTTATCTTTCTGTCCTGCGAGA |
| *IL1B* | CAACAAGTGGTGTTCTCCAT | GAGGTGCTGATGTACCAGT |
| *IL4* | GAAGCTGAGCAACTGTCCTG | TCTTGCATGGCGGTCTTTAG |
| *IL8* | TGGCTCTCTTGGCAACCTTC | CAGAACTGCAGCTTCACAAAGAGT |
| *KLF4* | AGAAGCATCTCGGGCAATTC | AGTCGCTTCATGTGGGAGAG |
| *LGR5* | TAACTGGAACTGCGAACCTG | AGCTAAATTCAGGGACCGGA |
| *LYZ* | AGGTGTGAGTTGGCCAGAAC | ACCAGTAGCGGCTATTGATCT |
| *MKI67* | TGGAGTAATCTATGTGGGCCA | CACCAAGAGCCTTTCACCAAA |
| *MCT1* | GGCTTCCTTCTGCAATACCG | ACACAGCAGTTCAGTAGGCA |
| *MUC1* | GCGCTGGCCATCATCTATTT | GCCCTGGTGAATGACATGTC |
| *MUC13* | TTTGGCTACAGTGGAGTGGG | TGGTTTGCGTAGGGGTTTTG |
| *MUC2* | AGCGATGACTTCAGGACGGC | GTCGTGACAGCTGGACTGGG |
| *MYD88* | GGATGGTGGTGGTCGTCTC | TTCTTCATGGCCTTGTACTTGA |
| *NOS2* | GAACTCTCAGCTCATCCGGT | CACCTCGAGCACAATGTCAG |
| *NOX1* | GGTCACTCCCTTTGCTTCCA | TATTGCTGCATGGCCAACAA |
| *NP4* | GGATGAAACCTCCCCTCTTGA | ATTGACTGTGCAGGACCCAG |
| *NRF2* | AATTCGCCGACTCTGACTCT | TTTTCGAGGACTGGGACTCC |
| *OCLN* | TGGAATGACGGGTCTCTACG | CGGTGTGGACTTGTAGGACT |
| *PCNA* | GCCGAGACCTCAGCCATATT | TGTCCCATGTCGGCAATTTT |
| *PIGR* | GGAGCCATTGACAACCCAAG | CTTGGGCTCCTCAATCTCCA |
| *PLA2G4A* | CGTCAAGCCCGACGTCTCTG | GCACTTCCCCAGACACCCAT |
| *PTGS2* | TACGGTGAAAACTGCTCCAC | GAGGTTAGAGAAGGCTTCCCA |
| *REG3G* | ATGGACATGGATGGGAGTGG | GGGTAACTGCGCATCACAAT |
| *S100A8* | AGGAAATTACCACGCCCTCT | CGGACACGCCTATCTTCACA |
| *S100A9* | GAGGACCTGGACACGAACC | GGAGGCTTTATTTTCCCCACC |
| *SOD1* | GATTCCATGTCCACCAGTTTG | CCTTTGCCCAAGTCGTCTTC |
| *TGFB1* | GTGGACAGCAACAACAAAATCT | CAACTCCAGTGACATCGAAGG |
| *TJP1* | GACCACTCCAGACAGTCTCC | CTGAGTTAGGCAGGACACCA |
| *TJP2* | CAGGGTGGTCATGGTTAACG | GCCTGCTCCTCTCACTGTAT |
| *TLR2* | GATGGATTTTGCCGGAGTCC | TCGTAGCTCTGCAGGTTTGA |
| *TLR4* | CATGTCTCAGAACTGCACTTTC | CCACAGCCACTAGTTTCTGC |
| *TLR5* | ACCTCGCCCACAACAAGATA | GGGATCTGAATGTTTGGGCC |
| *TNFA* | GGAGGAAGAGTCCCCAAACAA | ATCTGGGCCACAGGGTTGA |
| *TNFSF13* | CACTTCTTCACCTTGTTCCCA | CCGGGGAATTACGACACTCA |
| *TNFSF13B* | AGGAAGAAGCGTGCCGTT | AGTACCCGGTTTCTTTGACC |

**SUPPLEMENTAL FIGURES**

Supplemental figure 1: **Identification of metabolites in caecal content NMR spectra.** Peaks are identified with a number corresponding to the metabolites described in supplemental table 2. The spectra are representative of caecal content of rabbits exclusively suckling (A, C) or after the onset of solid food ingestion (B, D). The aliphatic (A,B) and aromatic (C,D) regions are shown.
