# Supplementary figures and images for: Gut microbiota derived metabolites contribute to intestinal barrier maturation at the suckling-to-weaning transition
Source: Gut Microbes. 2020 Apr 30;11(5):1268–86. doi: 10.1080/19490976.2020.1747335 (PMC7524271; doi:10.1080/19490976.2020.1747335)

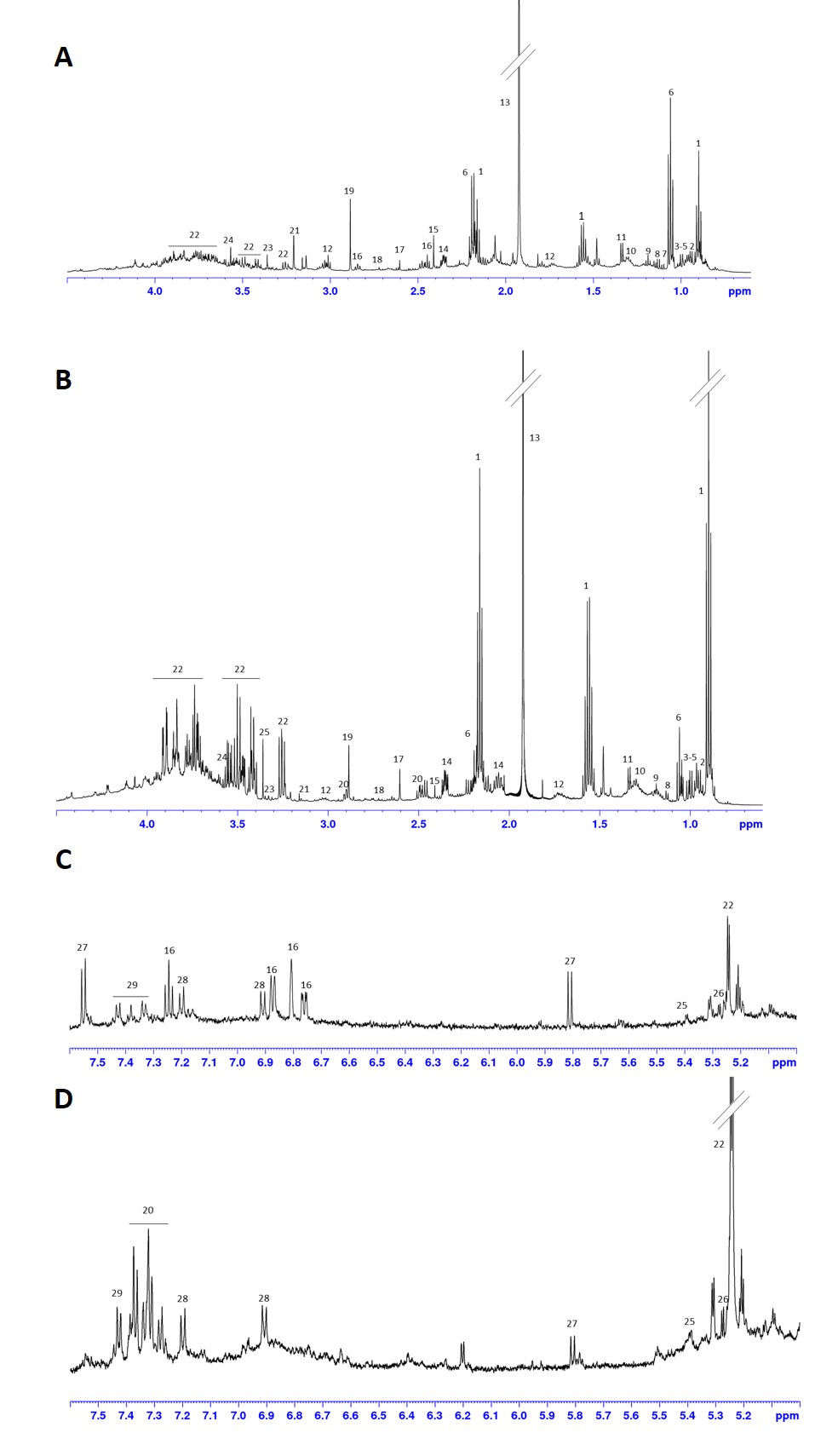

Supplement: Supplemental Material [file KGMI_A_1747335_SM6366.zip › Supplementary information/Figure_supp_1.jpg]
